# A Novel Design Concept of Cemented Paste Backfill (CPB) Materials: Biobjective Optimization Approach by Applying an Evolved Random Forest Model

**DOI:** 10.3390/ma15238298

**Published:** 2022-11-22

**Authors:** Yanjun He, Yunhai Cheng, Mengxiang Ma, Fenghui Li, Yaxin Song, Long Liu, Xudong Wang, Jiandong Huang

**Affiliations:** 1 Lijiahao Coal Mine, Baotou Energy Co., Ltd., China Energy Investment Corporation, Ordos 017008, China; 2 College of Resources, Shandong University of Technology, Qiangangwan Road, Qingdao 100027, China; 3 School of Mining Engineering, Anhui University of Science and Technology, Huainan 232001, China; 4 School of Civil Engineering, Guangzhou University, Guangzhou 510006, China

**Keywords:** uniaxial compressive strength, cost, multiobjective optimization model

## Abstract

For cemented paste backfill (CPB), uniaxial compressive strength (UCS) is the key to ensuring the safety of stope construction, and its cost is an important part of the mining cost. However, there are a lack of design methods based on UCS and cost optimization. To address such issues, this study proposes a biobjective optimization approach by applying a novel evolved random forest (RF) model. First, the evolved RF model, based on the beetle search algorithm (BAS), was constructed to predict the UCS of CPB. The consistency between the predicted value and the actual value is high, which proves that the hybrid machine learning model has a good effect on the prediction of the UCS of CPB. Then, considering the linear relationship between the costs and the components of CPB, a mathematical model of the cost is constructed. Finally, based on the weighted sum method, the biobjective optimization process of the UCS and cost of CPB is conducted; the Pareto front optimal solutions of UCS and the cost of CPB can be obtained by the sort of solution set. When the UCS or the cost of CPB is constant, the Pareto front optimal solutions can always have a lower cost or a higher UCS compared with the actual dataset, which proves that the biobjective optimization approach has a good effect.

## 1. Introduction

With the increase in demand for mineral products, the scale of mining development is also expanding [1,2]. However, the exploitation of mineral resources has caused serious environmental pollution and ecological damage [3,4]. The increase of tailings accumulation not only occupies a large amount of land and the storage investment is huge, it also causes certain pollution to the surrounding ecological environment, and the tailings contain a large number of useful metals and nonmetallic minerals, which if not used are a waste of resources [5,6,7]. Therefore, the exploitation and utilization of tailings resources is beneficial to accelerate the development of the economic cycle, promote an energy-savings and emissions-reduction system, and facilitate the implementation of a sustainable production mode [7,8,9]. Making cemented paste backfill (CPB) from tailings as filling aggregate for underground mining areas is the most commonly used means to treat tailings [10,11,12,13]. CPB refers to a kind of cement-based material that is set and solidified by mixing dehydrated tailings, cementitious materials, water, etc., in a certain proportion [14,15,16]. Filling underground mining areas with CPB by specific methods can not only efficiently treat tailings, it also helps to reduce surface subsidence, improve the underground environment of mining operations, and enhance ore recovery [17,18,19]. The UCS of CPB is the key to ensuring the safe implementation of backfill mining [20,21]. To make better use of the UCS of CPB for mining operations, many researchers have studied it. Fu et al. [22] studied the influence of solid mass fraction, cement-to-sand ratio, and curing time on the UCS of CPB. The results of X-ray diffraction (XRD) and scanning electron microscope have shown that the UCS of CPB increased with the increase of ettringite and C-S-H gelling agent, and the UCS of CPB is proportional to the solid mass fraction, cement, and curing time. The above research results show that engineers can enhance the UCS of CPB to ensure the safety of backfill construction by increasing the solid mass fraction, cement, and curing time. Jiang et al. [23] determined the effects of the parameters, such as the water-binder ratio (W/B), activator dosage, adhesive content, and sodium silicate/sodium hydroxide (SS/SH) on the UCS of AAS-CPB (AAS-CPB). The results also showed that the UCS of AAS-CPB was more sensitive to SS/SH and depended on the curing time, the UCS of AAS-CPB increased with the decrease of W/B and with the increase of binder content, and if the dosage of the activator was low, the UCS of AAS-CPB would increase as the activator dosage increased. Xu et al. [24] studied the relationship between the UCS of CPB and several important design parameters during the hydration process based on electrical resistivity (ER) measurement. The conclusion showed that the UCS of CPB increased with the increase of any variable of cement to tailings ratio, solid content, and curing time. The ER characteristics of CPB samples were highly correlated with their respective microstructural properties, and the ER test can effectively preliminarily predict the UCS of CPB. This study provides a new idea for CPS prediction. Yu et al. [25] proposed a new artificial intelligence model to predict the UCS of fiber-reinforced CPB and established a large and reliable database to evaluate the reliability of the model. The use of an artificial intelligence model to evaluate the UCS of CPB has achieved good results and can effectively overcome the shortcomings of the traditional laboratory test method, such as high cost, long time, etc. However, it should be noted that these previous studies are only focused on the modeling of the mechanical properties of the CPB and only employed a laboratory test, while the lack of evolutionary algorithms to optimize the prediction process and effect resulted in unsatisfactory modeling results [26,27].

The cost of CPB is an important part of the mining cost, accounting for about 20% of the mining cost [6,21,28]. Therefore, since the cost of CPB is of great significance for reducing the mining cost, some researchers have carried out a series of studies on the price of CPB [28,29]. Bian et al. [30] studied fiber-reinforced CPB to reduce the overall usage and cost of cement, and improve the mechanical properties of CPB. It is indicated that the yield stress of CPB decreased continuously with the increase of initial sulfate content, while the viscosity increased. Jin et al. [31] analyzed the influence of polyacrylamide (PAM) and polycarboxylate superplasticizer (PCE) on CPB with varying water/solid ratios W/S and W/C. The results showed that the high W/C (1.0 and 1.2), low W/S (0.3), 0.05% PAM, and 1.0% PCE can benefit the production cost as well as the physical properties of the CPB. Chang et al. [32] proposed the use of fly ash in CPB to reduce the costs and improve the strength of backfill. The research results show that replacing cement in CPB with fly ash can effectively reduce its cost and have an overall impact on the strength of it.

**Figure 1:**
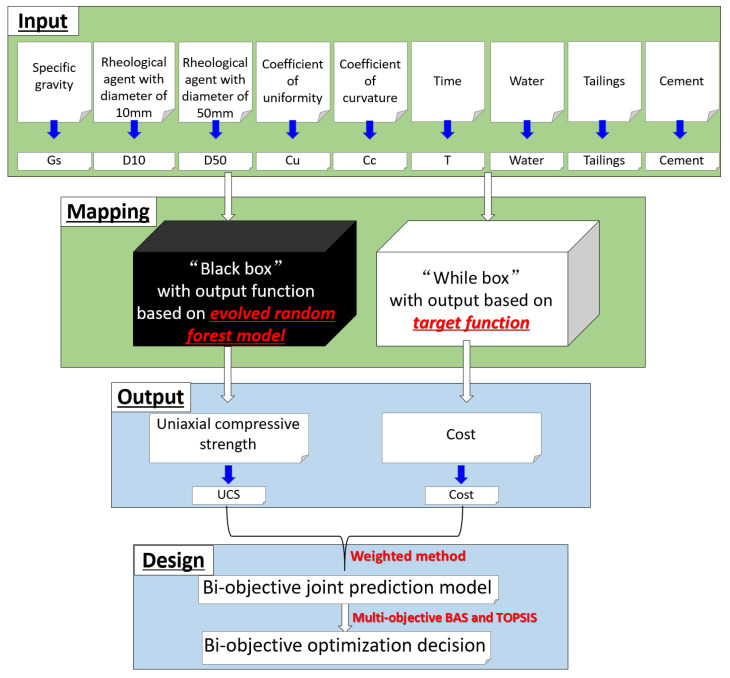
Research process.

Although some researchers have studied the UCS and the cost of CPB, and achieved certain research results, few researchers have optimized the UCS and the cost of CPB at the same time [33,34,35], while it should be noted that the coal back-filling engineers tended to be cautious about the cost. Also, the use of intelligent combinatorial algorithms to predict the performance of CPB is missing in past research. Researchers usually use the laboratory test method to study the performance of CPB [3,10,12,36]. However, the laboratory test method has many shortcomings, such as high cost, long time, and high labor consumption. Especially when studying the impact of multiple variables on the performance of CPB, the number of test pieces to be prepared will increase exponentially, and the corresponding cost and time will also increase exponentially. To address these challenging issues in the CPB design process, the present study develops a biobjective optimization model for the UCS and cost optimization of CPB. First, to improve the reliability and efficiency of the prediction results, a new evolutionary algorithm is proposed in this paper. In such a process, the so-called beetle search algorithm (BAS) was employed to optimize the random forest (RF) hyperparameters, and the evolved RF was employed to model the UCS of CPB [37,38,39]. Then, according to the linear relationship between the CPB mixture and the cost, the mathematical model of CPB cost optimization was established, and the multiobjective optimization problem was transformed into a single objective optimization problem by using a so-called weighted sum method. Finally, the optimal Pareto front solution set is obtained by using the order preference by similarity to the ideal solution (TOPSIS). The research process of the present study can be summarized in Figure 1.

## 2. Methodology

### 2.1. Optimization of the UCS for CPB

#### Data Analysis

**Figure 2:**
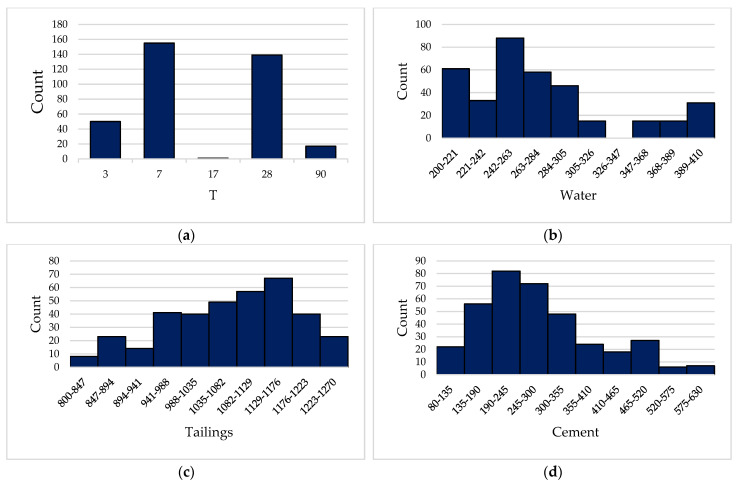

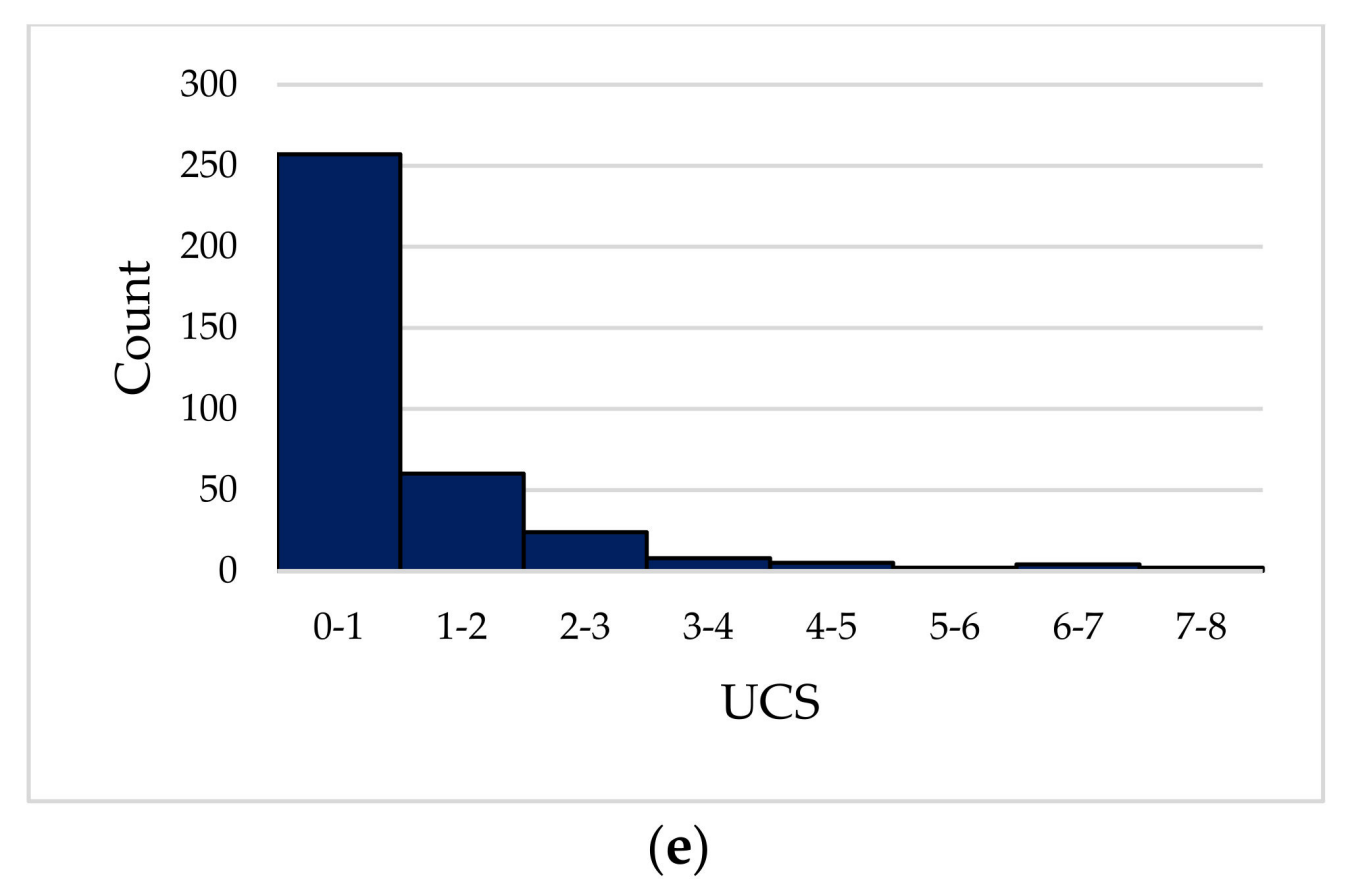
Frequency distribution histogram of input parameters ((**a**): T; (**b**): Water; (**c**): Tailings; (**d**): Cement; (**e**): UCS).

To ensure the verification of the accuracy of the established model, a reliable database containing 362 datasets was established in this study, and the datasets in the database were collected from published literature. As shown in Figure 1, the UCS of CPB is the output variable and the input variables of the database are the specific gravity (Gs), the rheological agent with a diameter of 10 mm(D10), the rheological agent with a diameter of 50 mm(D50) (as can be seen from the previous research literature, the diameters of rheological agents commonly used in CPB are 10 mm and 50 mm [40,41,42], so this study uses these two diameters of rheological agents as the two input variables for predicting the UCS of CPB), the coefficient of uniformity (Cu), coefficient of curvature (Cc), time (T), water, tailings, cement, among which Gs, D10, D50, Cu, and Cc are indicated in the physical properties of the tailings. According to its physical properties, it can be divided into thirteen different kinds, as shown in Table 1. T, water, tailings, cement, and the UCS of frequency distribution histogram are presented in Figure 2. The binder content is varying from 88 kg to 629 kg for one cubic meter of CPB. The input variables T, water, tailings, and cement have wide numerical coverage and reasonable settings, so the output variable has reasonable numerical distribution and wide coverage.

**Figure 3:**
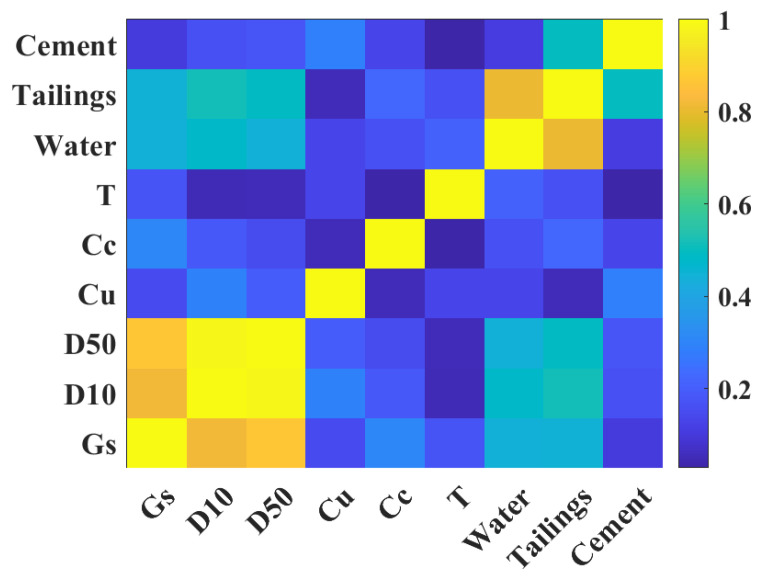
Correlation analysis between input variables.

Before the start of model training, it is necessary to analyze the correlation between the input variables, because it can determine whether the selected input variables can accurately predict the UCS of CPB [43,44]. In this study, SPASS software was used to analyze the correlation between input variables, and the results are presented in Figure 3. The overall correlation of the input variables is low, with only the correlations between DS and D10, and D50, D10, and D50, as well as the tailings and water that are higher than 0.6. The correlations between the other input variables are lower than 0.6. It indicates that the nine input variables determined in the present research are reasonable to predict the UCS of CPB, and the prediction effect of the model will not be affected because of the high correlation between input variables.

### 2.2. Optimization Model of UCS for CPB

#### 2.2.1. Beetle Search Algorithm (BAS)

The employed BAS is an intelligent optimization algorithm inspired by the principle of beetle foraging [45,46,47]. The principle is thus: the beetle receives the smell of food through whiskers. If the left side receives the strong smell of food, the beetle moves to the left, otherwise, it moves to the right, according to this principle, until the beetle finds the food. The search process of BAS can be described by the following steps.

Step 1: Initialization parameters. The K-dimensional optimization problem, x represents the center of mass, $x_{l}$ and $x_{r}$ represent the left and right of the beetle’s whiskers, d represents the initialization parameters, and δ represents the initial step size.

Step 2: Randomly generate the K-dimensional vector and normalize them to unit vectors, the formula is as follows:

(1) $$b=\frac{rand(k,1)}{\left\|rand(k,1)\right\|}$$

where, rand(k,1) represents a *k*-dimensional random vector, and the left and right whiskers should be respectively expressed as:

(2) $$x_{l}=x_{t}+\frac{d_{t}\cdot b}{2}$$

(3) $$x_{r}=x_{t}-\frac{d_{t}\cdot b}{2}$$

where $x_{t}$ represents the position of the centroid of the longhorn at the first iteration and $d_{t}$ represents the distance between the two whiskers at the tth iteration.

Step 3: Calculate the fitness values $f_{l}$ and $f_{r}$ of the left and right whiskers, and determine the direction of beetle advance according to the size relationship of the two whiskers:

(4) $$x_{t}=x_{t-1}-\delta_{t}bsign(f_{l}-f_{r})$$

where sign(⋅) represents the sign function and $\delta_{t}$ represents the step length of the beetle at the t iteration.

Step 4: Calculate the fitness value after the moving of the beetle, and update the distance between the left and right whiskers and the step length of the beetle.

(5) $$\delta_{t}=eta-\delta \cdot \delta_{t-1}$$

(6) $$d_{t}=eta-d\cdot d_{t-1}$$

**Figure 4:**
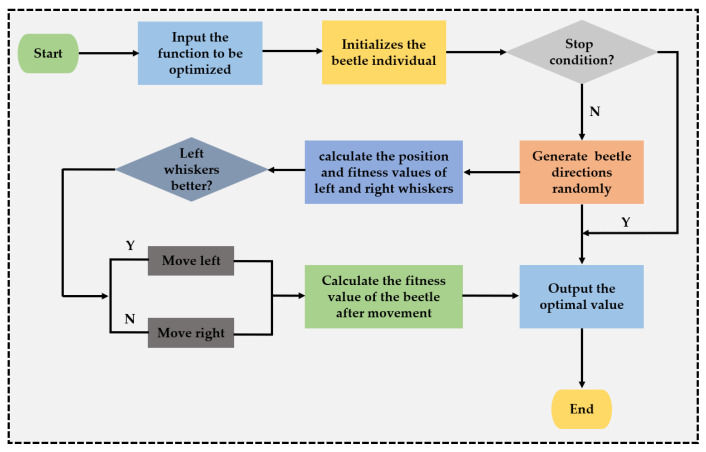
Introduction of BAS.

Figure 4 gives the process to determine the fitness value of the beetle after movement.

#### 2.2.2. Random Forests (RF)

**Figure 5:**
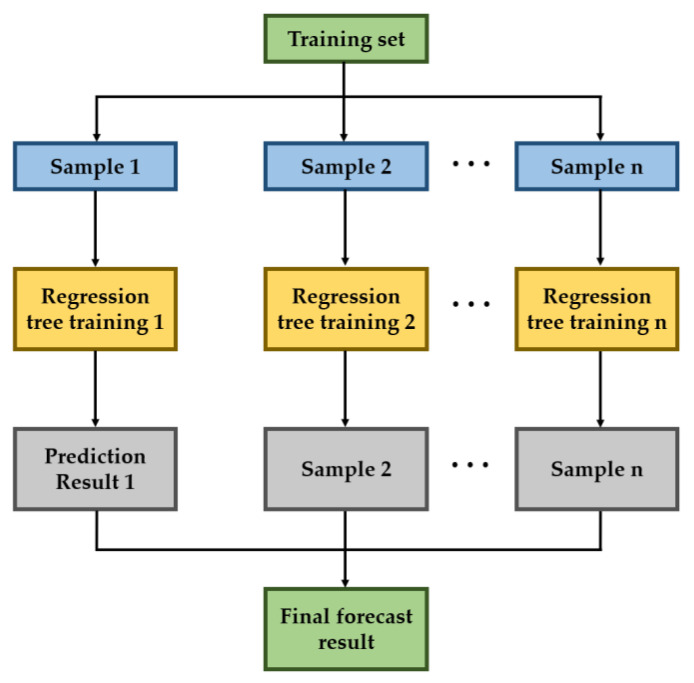
Flow chart of RF.

The so-called RF is based on the idea of a decision tree and Bagging ensemble learning, and the output result is the mode or average of multiple decision tree results. RF overcomes the shortcoming of a low prediction accuracy of the single decision tree and improves the applicability of the model. Figure 5 gives the program of the RF model.

The process of RF modeling is as follows:

(1) Data preprocessing. According to the requirements, the data set is divided into an input layer and an output layer. The employed dataset should be randomly divided into the training and testing parts according to the proportion.
(2) Operation of the model. The RF model is established by using the training data set, and the model results are output.
(3) Model evaluation. Select appropriate indexes and datasets to test the prediction effect of the established RF model on the UCS of CPB.
(4) Repeat steps (2) and (3) until the best model parameters and results are found.

**Figure 6:**
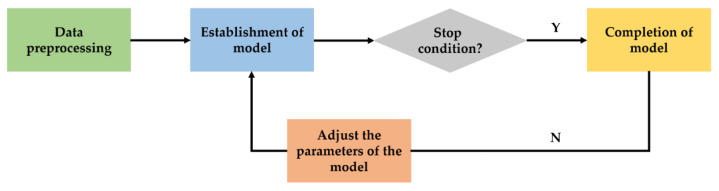
Flow chart of RF modeling.

The flow chart of RF is shown in Figure 6.

### 2.3. Optimization Model of Cost for CPB

There is an obvious linear relationship between the cost of CPB and its components. In this study, the cost of CPB is optimized by mathematical formula modeling; the formula is as follows:

(7) $$\mathrm{Cos}t=C_{W}Q_{W}+C_{T}Q_{T}+C_{C}Q_{C}$$

where $C_{W}$, $C_{T}$, and $C_{C}$, respectively, represent the costs of water, tailings, and cement; $Q_{W}$, $Q_{T}$, and $Q_{C}$, respectively, represent the quantities of water, tailings, and cement in each cubic meter of concrete; The unit price of water, tailings, and cement are 0.0024 $/kg, 0.02 $/kg, and 0.0475 $/kg; the densities are 1000 kg/m^3^, 2500 kg/m^3^ and 3150 kg/m^3^; thus the unit price for water, tailings, and cement are 0.24 $/m^3^, 50 $/m^3^, and 150 $/m^3^, respectively.

(1) Constraints of range

In specific optimization problems, the range of the decision variable values is determined by the range of corresponding variable values in the database:

(8) $$V_{i\min}\leq V_{i}\leq V_{i\max}$$

where $V_{i}$ represents the ith decision variable, w represents the minimum value of the ith decision variable, and $V_{i\min}$ represents the maximum value of the ith decision variable.

(2) Constraints of the mixture proportion

Regarding the mixture design process, it is very important to restrict the corresponding proportion according to the data in the database, mainly including the water-to-cement ratio ($\frac{W}{C}$), the water-to-solid ratio ($\frac{W}{C+T}$), and the tailings-to-cement ratio ($\frac{T}{C}$).

(9) $$0.31\leq \frac{W}{C}\leq 4.64$$

(10) $$0.10\leq \frac{W}{C+T}\leq 0.47$$

(11) $$1.27\leq \frac{T}{C}\leq 14.27$$

(3) Volume constraint

The total volume of each component in CPB is one, and the constraints are as follows:

(12) $$V_{m}=\frac{Q_{w}}{W_{w}}+\frac{Q_{T}}{W_{T}}+\frac{Q_{C}}{W_{C}}$$

where $W_{W}$ represents the unit weight of water, $W_{T}$ represents the unit weight of tailings, and $W_{C}$ represents the unit weight of cement.

(4) Constraints on the UCS of CPB

According to the actual conditions, the UCS of the optimized CPB must be greater than the design value, and there needs to be an upper bound, the UCS range constraint of the optimized CPB is as follows:

(13) $$P_{cg}\leq P_{c}\leq P_{cg}^{\prime }$$

where $P_{cg}$ is the given UCS, $P_{c}$ is the predicted UCS, and $P_{cg}^{\prime }$ is the maximum value of the UCS.

### 2.4. Biobjective Optimization Model Considering the UCS and Cost

**Figure 7:**
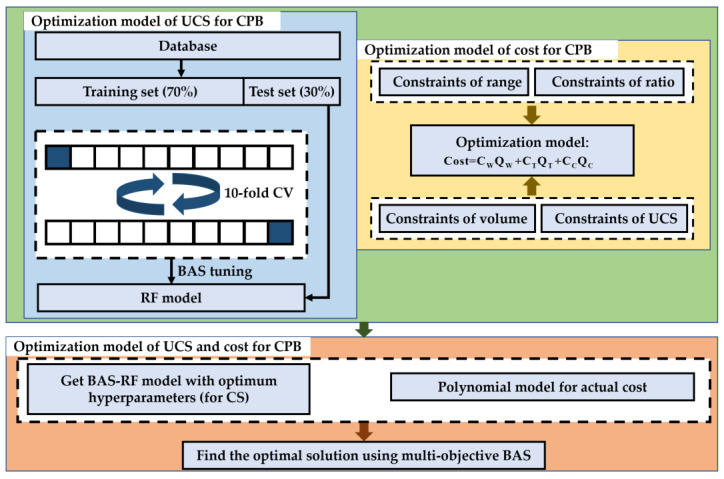
Flow chart for the optimal design of CPB mixtures.

In the present study, the biobjective optimization model including the UCS and cost of CPB was transformed into the single objective one by using the weighted sum method. Then, the solution set with a minimized objective function is searched. Finally, regarding the UCS and the cost for the CPB, the Pareto frontier optimal solution set can be obtained by the following process (Figure 7).

#### 2.4.1. Biobjective Problem

The multiobjective optimization problem refers to when there are two or more optimization objectives, and these objectives are coupled together by decision variables in a competitive state so they cannot be optimized to the best at the same time. Compared with the single objective extremum solution, the multiobjective optimization solution is more complex and exists more widely in real life [48]. Usually, the maximization problem is transformed into the minimum problem by taking the reciprocal or negative value. The general formula of the biobjective optimization solution can be obtained by the equations below.

(14) $$\min F(X)=(f_{1}(x),f_{2}(x),\cdots f_{m}(x))$$

(15) $$G(X)=(g_{1}(x),g_{2}(x),\cdots ,g_{k}(x))\leq 0$$

(16) $$H(X)=(h_{1}(x),h_{2}(x),\cdots h_{j}(x))=0$$

in which X represents the decision variable, F(X) is the target solution to be optimized, G(X) and H(X) present the inequality and equality constraint, respectively.

#### 2.4.2. Pareto Optimality

**Figure 8:**
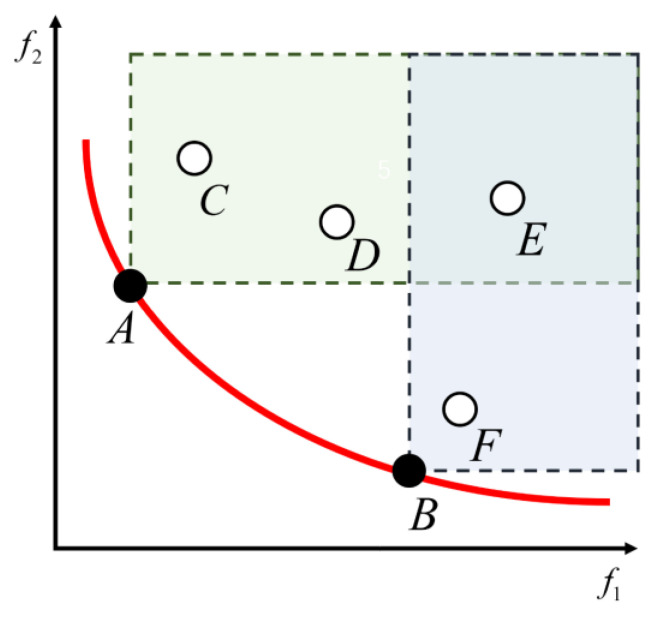
Schematic diagram of Pareto dominance relation (A and B: Pareto non-dominated solution; C, D, E, and F: Dominated solution).

Regarding the process to obtain the single objective optimization solution, the merits of the corresponding decision variable are evaluated directly by comparing the value of the two solutions. Regarding the process to obtain the solution of the multiobjective optimization, there are multiple objective functions, so the merits of decision variables cannot be evaluated simply by comparing the values of functions. To address such issues, the concept of Pareto domination is employed in the present study, the core idea of which is to judge the merits of the solution by comparing the value of the decision variable at the corresponding position. In the optimization problem of minimizing the optimal, assuming that $x_{1}$ and $x_{2}$ for all subtargets there $f(x_{1})\leq f(x_{2})$, that is to say, all corresponding subtargets’ corresponding solutions of $x_{1}$ are lower than (or equal to) the subtargets function value of $x_{2}$, and that there are at least corresponding objective function values of $x_{1}$, which is less than the sub-targets function value of $x_{2}$. This means that $x_{1}$ is dominant over $x_{2}$. In the process of obtaining the solution of the multiobjective optimization, it is typically conflicted between the target variables; if the target improves to a subtarget, it is likely to the optimization effect of other subtargets, so the optimal solution of multiobjective optimization problems usually is not the only solution, but made up of multiple nondominated solutions of the optimal solution set. Regarding the biobjective optimization problem in this study, the Pareto dominance relations are shown in Figure 8, considering that the minimum objective function is optimal.

#### 2.4.3. Weighted Sum Method

In the present research, the biobjective (UCS and cost) optimization problem was addressed by using the so-called weighted sum method. It gives weight coefficients to different subobjective functions and then forms new objective functions, transforming the biobjective optimization to one with only a single objective by the following equation.

(17) $$f=\sum_{i=1}^{m}\omega_{i}f_{i},\sum_{i=1}^{m}\omega_{m}=1$$

in which $\omega_{i}$ represents the weight of the first (UCS) and second objective (cost), and $\omega_{i}\in \left[0,1\right]$.

#### 2.4.4. Decision-Making Method

In this study, the technique for order preference by similarity to an ideal solution (TOPSIS) was employed as the decision-making method [49]. It is an evaluation method approximating the ideal solution ranking, which requires the function to be monotonical (monotonically increasing or monotonically decreasing). This method can make full use of the information in the original data to accurately reflect the gap between evaluation schemes. The basic principle of TOPSIS is to sort the detected objects by calculating the distance between them, the optimal solution, and the worst solution. If the evaluation objects are closer to the optimal solution, and at the same time are furthest away from the worst solution, the solution is the optimal one, and vice versa is the least optimal one. The calculation formula for the positive ideal solution ($d_{i+}$), negative ideal solution ($d_{i-}$), and proximity coefficient ($C_{i}$) are as follows:

(18) $$d_{i+}=\sqrt{{\sum_{j=1}^{n}\left(F_{ij}-F_{j}^{ideal}\right)}^{2}}$$

(19) $$d_{i-}=\sqrt{{\sum_{j=1}^{n}\left(F_{ij}-F_{j}^{non-ideal}\right)}^{2}}$$

(20) $$C_{i}=\frac{d_{i-}}{d_{i+}+d_{i-}}$$

where i represents a Pareto solution, n represents the number of objectives, $F_{j}^{ideal}$ represents the jth ideal solution in the single objective optimization problem, $F_{j}^{non-ideal}$ represents the jth nonideal solution in the single objective optimization problem, and $C_{j}$ is the proximity coefficient. The larger it is, the better the corresponding solution is, the smaller it is, the worse the corresponding solution is.

## 3. Results and Discussion

### 3.1. Hyperparameter Tuning

**Figure 9:**
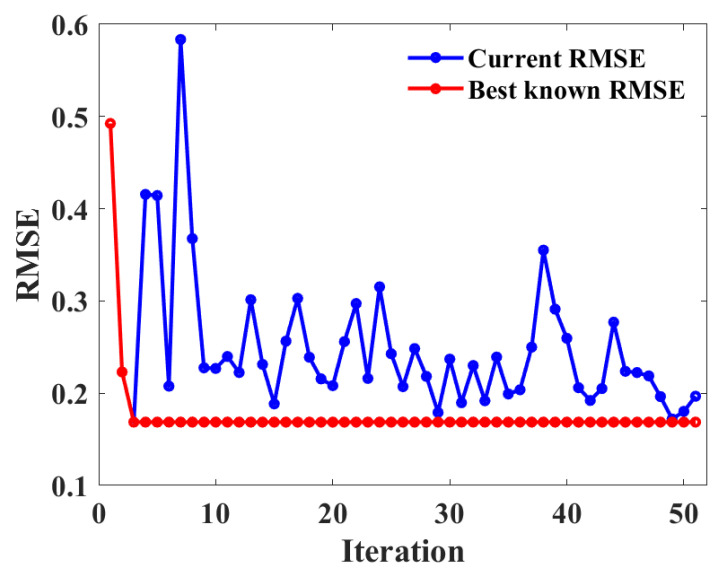
Relationship between RMSE value and iteration number.

**Figure 10:**
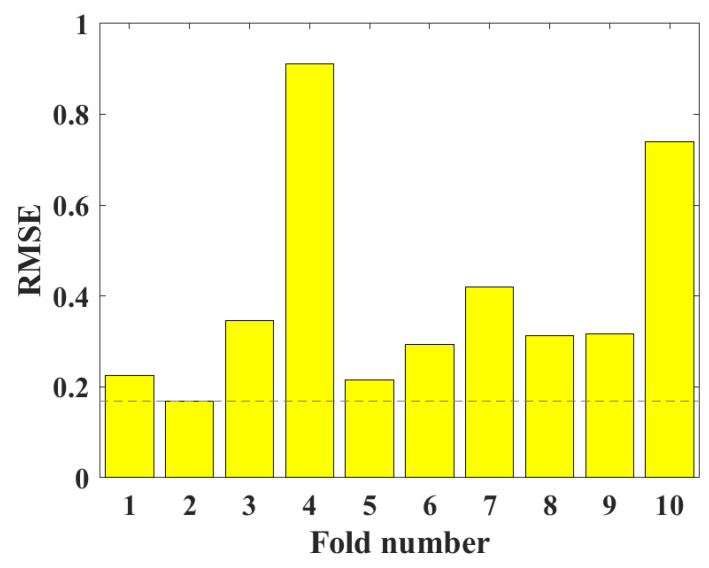
Ten-fold CV for hyperparameter tuning on the UCS dataset of CPB.

In this study, BAS and 10-fold CV were used to optimize the RF model to predict the UCS of CPB. Figure 9 shows the relationship between the number of iterations and the root mean square error (RMSE) value. It can be clearly seen from the figure that with the increase in the number of iterations, the RMSE value first converges sharply to a lower value and then tends to be stable. When the number of iterations reached 10, the RMSE value decreased to the minimum. In order to obtain the optimal hyperparameters, a 10-fold CV was used to optimize the RF hyperparameters. As shown in Figure 10, the minimum RMSE value is obtained at the second fold. The above analysis results show that BAS and 10-fold CV have a good tuning effect on RF hyperparameters.

### 3.2. Evaluation of the Optimization Model of the UCS

**Figure 11:**
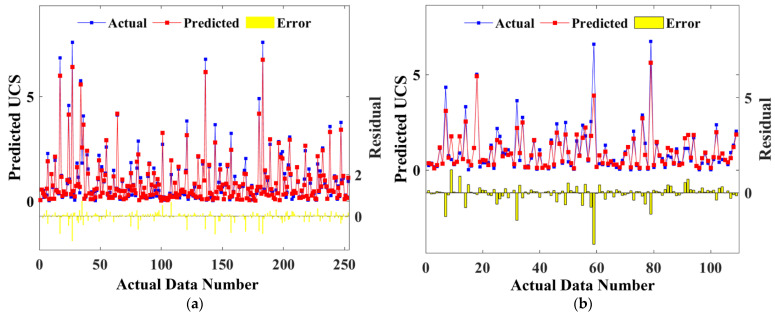
Comparison of predicted and actual values for training sets (**a**) and test sets (**b**).

Figure 11 shows the comparison between the predicted UCS values of the training data set and the actual UCS values of the test data set CPB. The horizontal lines in the figure represent errors. It can be seen that the predicted values of the training set and the test set have a high consistency with the measured values. There are only a few points with large errors in the test set, however, these points with large errors will not affect the model’s prediction effect on the UCS of CPB.

**Figure 12:**
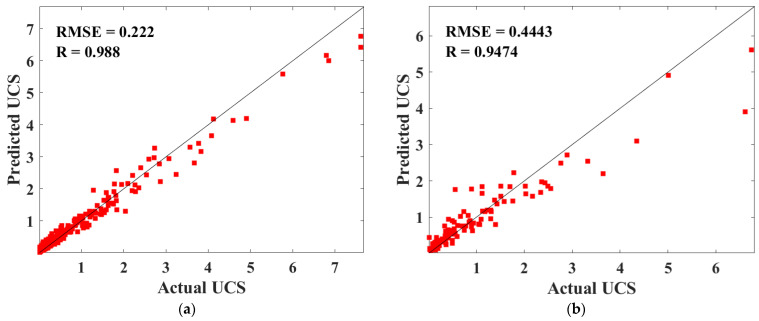
Predicted and actual values for training sets (**a**) and test sets (**b**).

Figure 12 shows the fitting effect between the predicted UCS values of CPB in the training and testing datasets. From the figure, it can be indicated that the predicted and actual values of the training and test sets fit well, and these points are close to the perfect fitting curve with R = 1. The R values of the training set and the test set were 0.988 and 0.9474, respectively, and the RMSE values were 0.222 and 0.4443, respectively. The above analysis results prove that RF has a high prediction accuracy for the UCS of CPB.

**Figure 13:**
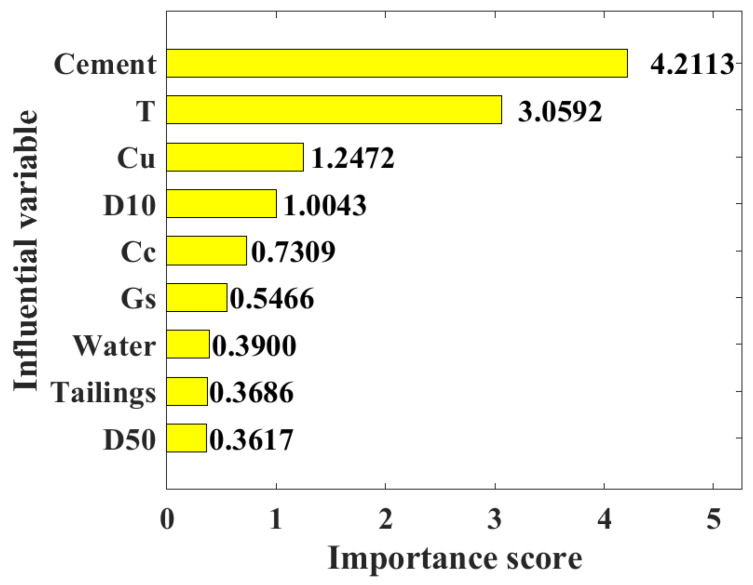
Importance score of the input variable.

To further determine the influence of each input variable on the UCS of the CPB, this study analyzed the importance scores of different input variables, and the results are shown in Figure 13. It is indicated from the figure that the importance scores of cement, T, Cu, D10, Cc, Gs, water, tailings, and D50 to the UCS of the CPB decrease successively; that is, cement and T have high sensitivity to the UCS, while tailings and D50 have low sensitivity. It should be noted that the important scores are based on the influence of varying input parameters on the UCS and these obtained scores will not be limited by any restriction.

### 3.3. Determination of the Effect of the Biobjective Optimization Model

**Figure 14:**
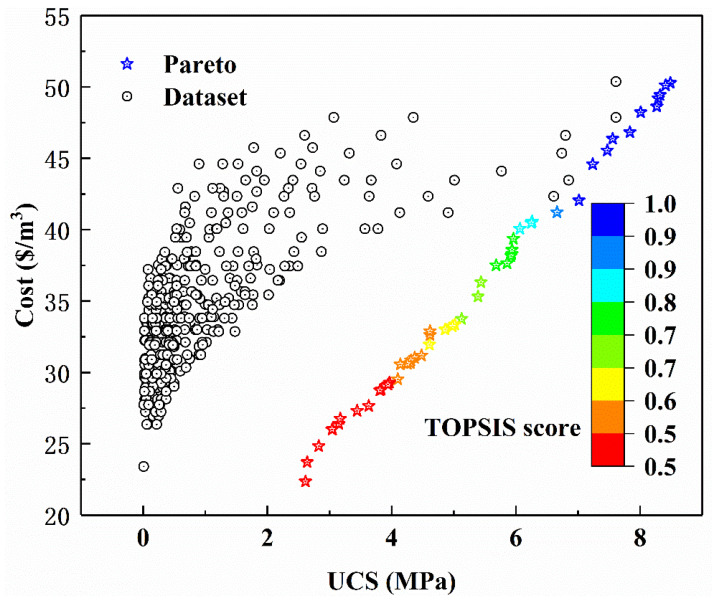
TOPSIS score of the input variable.

Figure 14 gives the results of the biobjective optimization regarding the UCS and the cost of CPB. It can be seen that the actual UCS of CPB have reasonable numerical distribution and wide coverage, and are all located above the Pareto front optimal solution of nondominant points. It is proved that the cost of the actual data set to reach a specific UCS is greater than the cost of the Pareto front-end optimal solution to reach the UCS, or the UCS of the actual data is smaller than the UCS of the corresponding Pareto front end optimal solution under the same cost. The above analysis proves that the biobjective optimization model proposed in this paper can effectively reduce the cost without weakening the mechanical properties, and effectively enhance the mechanical properties without increasing the cost. That is, the model has a good optimization effect on the cost and UCS as well as strong adaptability.

According to the above analysis, cement is the most sensitive input variable for CPB UCS, so the UCS of CPB can be improved by increasing the amount of cement added. However, due to the high cost of cement, increasing the amount of CPB will increase its cost. How to balance the cost of CPB and UCS is an important problem for researchers to solve. In this paper, the TOPSIS method is used to sort the optimal solutions of the Pareto frontier. It can be indicated from Table 2 that the highest TOPSIS score of the Pareto front optimal solution is 0.997, and the UCS of the corresponding CPB is 1.78 MPa, the cost is 45.75 $/m^3^, and the Gs, D10, D50, Cu, Cc, T, water, tailings, and cement corresponding to the mixture are 2.79 kg/m^3^, 0.08 kg/m^3^, 0.3 kg/m^3^, 4.65, 0.94, 7 days, 200 kg/m^3^, 1088 kg/m^3^, and 505 kg/m^3^, respectively.

## 4. Conclusions

The USC and cost optimization design of CPB is a challenging problem faced by the backfilling of goaf in the mining area. To solve this problem, this study established the UCS and cost prediction biobjective optimization model of CPB based on the machine learning model and mathematical formula modeling. Through the analysis of this study, the following points can be highlighted:

(1) BAS has good performance in RF hyperparameter tuning. The RMSE value of the UCS of CPB reaches the minimum value in the second iteration.
(2) The BAS and RF hybrid machine learning model achieves high prediction accuracy on the training set (R = 0.988, RMSE = 0.222 MPa) and test set (R = 0.9474, RMSE = 0.443 MPa) for the UCS of CPB.
(3) The sensitivity of different input variables to the UCS of CPB from high to low is cement, T, Cu, D10, Cc, Gs, water, tailings, and D50.
(4) The weighted sum method is used to transform the biobjective optimization problem of cost and UCS into a single objective optimization problem. Comparing the obtained Pareto front optimal solution set with the data set, it is found that under the same CPB cost, the Pareto front optimal solution set has a higher UCS, and under the same CPB UCS, the Pareto front optimal solution set has a lower cost. It is proved that the proposed biobjective optimization method is useful for the CPB optimization of the cost and UCS.
(5) The TOPSIS method was used to rank the optimal solution of the Pareto front optimal solution. The UCS, cost, and values corresponding to input variables of the top 20 Pareto front optimal solutions are listed.

The biobjective optimization approach provides an efficient solution for the optimization of the UCS and cost of CPB, and can also be used in the field of civil engineering to solve other biobjective optimization problems. In the future, researchers can focus on more performance optimization of CPB, not just the UCS and cost.

## Figures and Tables

**Table 1:** Physical characteristics of tailings.

Variables	1	2	3	4	5	6	7	8	9	10	11	12	13
Gs	2.08	2.08	2.61	2.67	2.71	2.75	2.79	2.83	2.83	2.83	2.86	2.91	2.91
D10	0.248	0.004	0.005	0.035	0.447	0.016	0.08	0.005	0.023	0.023	0.015	0.248	0.004
D50	1.442	0.012	0.083	0.195	0.244	0.084	0.302	0.049	0.192	0.192	0.012	1.442	0.019
Cu	8.3	4.65	27.43	7.42	11.645	6.56	4.65	12.7	10.54	10.54	12.8	27.43	6.57
Cc	0.9	0.87	0.87	1.07	1.084	0.9	0.94	1.3	2.04	2.04	1.33	2.04	0.91

**Table 2:** TOPSIS score of CPB mixture.

No.	Gs (kg/m^3^)	D10 (kg/m^3^)	D50 (kg/m^3^)	Cu	Cc	T (Days)	Water (kg/m^3^)	Tailings (kg/m^3^)	Cement (kg/m^3^)	UCS (MPa)	Cost ($/m^3^)	TOPSIS Score
1	2.79	0.08	0.3	4.65	0.94	7	200	1088	504	1.78	45.75	0.997
2	2.79	0.08	0.3	4.65	0.94	7	200	1133.33	420	1.3	42.66	0.994
3	2.79	0.08	0.3	4.65	0.94	7	200	1165.71	360	0.75	40.46	0.993
4	2.79	0.08	0.3	4.65	0.94	7	200	1208.89	280	0.38	37.53	0.989
5	2.79	0.08	0.3	4.65	0.94	7	200	1236.36	229.09	0.2	35.66	0.987
6	2.79	0.08	0.3	4.65	0.94	7	200	1255.38	193.85	0.18	34.36	0.982
7	2.79	0.08	0.3	4.65	0.94	28	200	1088	504	2.73	45.75	0.976
8	2.79	0.08	0.3	4.65	0.94	28	200	1133.33	420	1.83	42.66	0.968
9	2.79	0.08	0.3	4.65	0.94	28	200	1165.71	360	1.33	40.46	0.961
10	2.79	0.08	0.3	4.65	0.94	28	200	1208.89	280	0.85	37.53	0.943
11	2.79	0.08	0.3	4.65	0.94	28	200	1236.36	229.09	0.21	35.66	0.927
12	2.79	0.08	0.3	4.65	0.94	28	200	1255.38	193.85	0.2	34.36	0.903
13	2.08	0.248	1.44	8.3	0.9	3	200	1208.89	280	0.25	37.53	0.883
14	2.08	0.248	1.44	8.3	0.9	3	200	1236.36	229.09	0.14	35.66	0.857
15	2.08	0.248	1.44	8.3	0.9	3	200	1255.38	193.85	0.09	34.36	0.788
16	2.08	0.248	1.44	8.3	0.9	7	200	1208.89	280	0.55	37.53	0.783
17	2.08	0.248	1.44	8.3	0.9	7	200	1236.36	229.09	0.29	35.66	0.779
18	2.08	0.248	1.44	8.3	0.9	7	200	1255.38	193.85	0.21	34.36	0.768
19	2.08	0.248	1.44	8.3	0.9	28	200	1208.89	280	1.84	37.53	0.743
20	2.08	0.248	1.44	8.3	0.9	28	200	1236.36	229.09	0.58	35.66	0.721

## Data Availability

The data presented in this study are available on request from the corresponding author.

## References

[1] Cheng Q., Guo Y., Dong C., Xu J., Lai W., Du B. (2021). Mechanical properties of clay based cemented paste backfill for coal recovery from deep mines. Energies.

[2] Cui L., Fall M. (2019). Mathematical modelling of cemented tailings backfill: A review. Int. J. Min. Reclam. Environ..

[3] Eker H., Bascetin A. (2022). Influence of silica fume on mechanical property of cemented paste backfill. Construct. Build. Mater..

[4] Grabinsky M., Thompson B., Bawden W. Evaluating cemented paste backfill plug strength and the potential for continuous pouring 1. Proceedings of the 13th International Symposium on Mining with Backfill (MINEFILL).

[5] He W., Zheng C., Li S., Shi W., Zhao K. (2021). Strength development monitoring of cemented paste backfill using guided waves. Sensors.

[6] Zhao Y., Taheri A., Karakus M., Chen Z., Deng A. (2020). Effects of water content, water type and temperature on the rheological behaviour of slag-cement and fly ash-cement paste backfill. Int. J. Min. Sci. Technol..

[7] Hu J., Zhao F., Ren Q., Kuang Y., Zhou T., Luo Z. (2019). Microscopic characterization and strength characteristics of cemented backfill under different humidity curing conditions. R. Soc. Open Sci..

[8] Ionescu D., Petrolito J., Dare A., Pentreath Z., Sonnberger L., Destech Publicat I. Assessment of the effect of different cementing materials on the strength of cemented paste backfill. Proceedings of the 3rd International Conference on Green Materials and Environmental Engineering (GMEE).

[9] Jafari M., Shahsavari M., Grabinsky M. (2020). Experimental study of the behavior of cemented paste backfill under high isotropic compression. J. Geotech. Geoenviron. Eng..

[10] Li B., Zhang J., Yan H., Liu H., Zhu C. (2022). Thermal enhancement of gangue-cemented paste backfill with graphite and silica sand: An experimental investigation. Environ. Sci. Pollut. Res..

[11] Li B., Zhang J., Yan H., Zhou N., Li M. (2022). Experimental investigation into the thermal conductivity of gangue-cemented paste backfill in mine application. J. Mater. Res. Technol..

[12] Li G., Deng G.-z., Ma J. (2022). Numerical modelling of the response of cemented paste backfill under the blasting of an adjacent ore stope. Construct. Build. Mater..

[13] Yu X., Tang W., Li N., Jiang M., Huang J., Wang D. (2022). Refined decomposition: A new separation method for rap materials and its effect on aggregate properties. Construct. Build. Mater..

[14] Li Z., Shi X., Chen X. (2022). Effect of rice straw on tensile properties of tailings cemented paste backfill. Appl. Sci..

[15] Liu H.-l., Hou C., Li L., Du J.-f., Yan B.-x. (2021). Experimental investigation on flow properties of cemented paste backfill through l-pipe and loop-pipe tests. J. Cent. South Univ..

[16] Niu H., Hassani F.P., Kermani M.F., He M. (2021). Rheological and mechanical properties of fibre-reinforced cemented paste and foam backfill. Int. J. Min. Reclam. Environ..

[17] Tuylu S. (2022). Effect of different particle size distribution of zeolite on the strength of cemented paste backfill. Int. J. Environ. Sci. Technol..

[18] Wang Z., Wang Y., Wu L., Wu A., Ruan Z., Zhang M., Zhao R. (2022). Effective reuse of red mud as supplementary material in cemented paste backfill: Durability and environmental impact. Construct. Build. Mater..

[19] Wu W., Xu W., Zuo J. (2021). Effect of inclined interface angle on shear strength and deformation response of cemented paste backfill-rock under triaxial compression. Construct. Build. Mater..

[20] Xu S., Suorineni F.T., Li K., Li Y. (2017). Evaluation of the strength and ultrasonic properties of foam-cemented paste backfill. Int. J. Min. Reclam. Environ..

[21] Huan C., Zhu C., Liu L., Wang M., Zhao Y., Zhang B., Zhang X. (2021). Pore structure characteristics and its effect on mechanical performance of cemented paste backfill. Front. Mater..

[22] Fu J.-X., Song W.-D., Tan Y.-Y. (2016). Study on microstructural evolution and strength growth and fracture mechanism of cemented paste backfill. Adv. Mater. Sci. Eng..

[23] Jiang H., Han J., Li Y., Yilmaz E., Sun Q., Liu J. (2020). Relationship between ultrasonic pulse velocity and uniaxial compressive strength for cemented paste backfill with alkali-activated slag. Nondestruct. Test. Eval..

[24] Xu W., Tian X., Cao P. (2018). Assessment of hydration process and mechanical properties of cemented paste backfill by electrical resistivity measurement. Nondestruct. Test. Eval..

[25] Yu Z., Shi X.-z., Chen X., Zhou J., Qi C.-c., Chen Q.-s., Rao D.-j. (2021). Artificial intelligence model for studying unconfined compressive performance of fiber-reinforced cemented paste backfill. Transact. Nonferrous Met. Soc. China.

[26] Ma W., Tian Y., Zhao H., Orton S.L. (2022). Time-dependent behavior of reinforced concrete columns subjected to high sustained loads. J. Struct. Eng..

[27] Huang J., Shiva Kumar G., Ren J., Zhang J., Sun Y. (2021). Accurately predicting dynamic modulus of asphalt mixtures in low-temperature regions using hybrid artificial intelligence model. Construct. Build. Mater..

[28] Zhang B., Xin J., Liu L., Guo L., Song K.-I. (2018). An experimental study on the microstructures of cemented paste backfill during its developing process. Adv. Civ. Eng..

[29] Zhang B., Li K., Zhang S., Hu Y., Han B. (2022). A modeling method for predicting the strength of cemented paste backfill based on a combination of aggregate gradation optimization and lstm. J. Renew. Mater..

[30] Bian J., Fall M., Haruna S. (2021). Sulfate-induced changes in rheological properties of fibre-reinforced cemented paste backfill. Mag. Concr. Res..

[31] Jin J., Qin Z., Zuo S., Feng J., Sun Q. (2022). The role of rheological additives on fresh and hardened properties of cemented paste backfill. Materials.

[32] Chang B., Du C., Chu X., Zhang L. (2021). Study on the optimization of filling ratio and strength variation characteristics of cemented backfills containing fly ash. Front. Mater..

[33] Wang J., Zhang C., Fu J., Song W., Zhang Y. (2021). Effect of water saturation on mechanical characteristics and damage behavior of cemented paste backfill. J. Mater. Res. Technol..

[34] Qi C., Tang X., Dong X., Chen Q., Fourie A., Liu E. (2019). Towards intelligent mining for backfill: A genetic programming-based method for strength forecasting of cemented paste backfill. Miner. Eng..

[35] Mbonimpa M., Kwizera P., Belem T. (2019). Mine backfilling in the permafrost, part II: Effect of declining curing temperature on the short-term unconfined compressive strength of cemented paste backfills. Minerals.

[36] Wang Y., Yu Z., Wang H. (2021). Experimental investigation on some performance of rubber fiber modified cemented paste backfill. Construct. Build. Mater..

[37] Huang J., Zhang J., Li X., Qiao Y., Zhang R., Kumar G.S. (2022). Investigating the effects of ensemble and weight optimization approaches on neural networks’ performance to estimate the dynamic modulus of asphalt concrete. Road Mater. Pavement Des..

[38] Huang J., Zhang J., Gao Y., Liu H. (2021). Intelligently predict the rock joint shear strength using the support vector regression and firefly algorithm. Lithosphere.

[39] Huang J., Xue J. (2022). Optimization of svr functions for flyrock evaluation in mine blasting operations. Environ. Earth Sci..

[40] Celestin J.C.H., Fall M. (2009). Thermal conductivity of cemented paste backfill material and factors affecting it. Int. J. Min. Reclam. Environ..

[41] Jiang H., Fall M., Cui L. (2017). Freezing behaviour of cemented paste backfill material in column experiments. Construct. Build. Mater..

[42] Li J., Zhang C., Li L., Fan C., He Z., Qian Y. (2022). Utilization of low-alkalinity cementitious materials in cemented paste backfill of gold mine tailings. J. Renew. Mater..

[43] Huang J., Zhou M., Zhang J., Ren J., Vatin N.I., Sabri M.M.S. (2022). Development of a new stacking model to evaluate the strength parameters of concrete samples in laboratory. Iran. J. Sci. Technol. Transact. Civ. Eng..

[44] Huang J., Zhou M., Zhang J., Ren J., Vatin N.I., Sabri M.M.S. (2022). The use of ga and pso in evaluating the shear strength of steel fiber reinforced concrete beams. KSCE J. Civ. Eng..

[45] Kou B., Ren D., Guo S. (2022). Geometric parameter identification of medical robot based on improved beetle antennae search algorithm. Bioengineering.

[46] Liao L., Zhang F., Ieee Comp S.O.C. Beetle antennae search algorithm for community detection in complex network. Proceedings of the 16th International Conference on Computational Intelligence and Security (CIS).

[47] Zheng Q., Xiang D., Fang J., Wang Y., Zhang H., Hu Z. (2020). Research on performance seeking control based on beetle antennae search algorithm. Meas. Control.

[48] Chen D., Lv Z. (2022). Artificial intelligence enabled digital twins for training autonomous cars. Internet Things Cyber-Phys. Syst..

[49] Chen R., Shen H., Lai Y. (2022). A metaheuristic optimization algorithm for energy efficiency in digital twins. Internet Things Cyber-Phys. Syst..

